# HIRSUTISM: CLINICO-INVESTIGATIVE PROFILE OF 50 INDIAN PATIENTS

**DOI:** 10.4103/0019-5154.42387

**Published:** 2008

**Authors:** Nand Lal Sharma, Vikram K Mahajan, Rashmi Jindal, Mudita Gupta, Anju Lath

**Affiliations:** *From the Department of Dermatology, Venereology and Leprosy, Indira Gandhi Medical College, Shimla, India*

**Keywords:** *Acne*, *hyperandrogenism*, *polycystic ovarian disease*

## Abstract

**Background:**

Despite worldwide prevalence of hirsutism studies on hirsutism in Indian patients are not many.

**Aims:**

This retrospective study was carried out to assess the clinico-investigative profile of patients presenting with hirsutism.

**Materials and Methods:**

Medical records of 82 hirsutism patients diagnosed consecutively during July 2005 to October 2007 were analyzed.

**Results:**

The complete data of 50 patients aged between 13 and 47 years were available. Fifty percent patients were aged 20 to 30 years. The average F-G score was 10.3 ± 2.46. Associated signs of hyperandrogenism were acne (64%), oligomenorrhea or menstrual irregularities (36%), androgenetic alopecia (16%), acanthosis nigricans (6%) and seborrhea (4%). Polycystic ovaries were detected in 30% patients and 22% patients had elevated serum free testosterone levels. Family history of hirsutism was present in 18% patients.

**Conclusion:**

Hirsutism in Indian patients is not uncommon. Adolescent patients appear to be more concerned about hirsutism as compared to those in the older age group who were more often worried of late onset acne. All patients, however, were more concerned for facial hair than those on other body areas signifying that facial hair need to be given higher than current value in F-G score.

## Introduction

Hirsutism is defined as male-pattern growth of terminal body hair in women in androgen-stimulated locations such as face, chest, and areolae. Hirsutism can be classified broadly into 2 groups viz. androgen induced and non-androgen induced. Androgen induced can either be due to excessive endogenous androgen production (ovarian/adrenal) or exogenous due to drugs. Central over production of androgens, increased peripheral conversion of androgens, decreased metabolism and enhanced receptor binding are potential causes of hirsutism. Non-androgen induced hirsutism can be idiopathic, familial or drug induced.

Other accompanying signs and symptoms of hyperandrogenism include acanthosis nigricans, obesity, pelvic mass, signs or symptoms of virilization, features of Cushing's syndrome, acne, increased sebaceous activity and alopecia. The modified Ferriman-Gallwey (F-G) score^1^ is used to determine the severity of hirsutism by assessing the extent of hair growth in nine key anatomical sites. Simple laboratory measurement of total and free testosterone, dehydroepiandrosterone sulfate, and androstenedione identifies about half of the patients with hyperandrogenism.

Hirsutism is a frequent reason of cosmetic embarrassment, poor self esteem, and psychological distress for women world over. However, hirsutism has rarely been studied in Indian patients. In this communication, we report clinico-investigative profile of 50 patients of hirsutism diagnosed in the Dermatology Outpatient Department during July 2005 to October 2007.

## Materials and Methods

Medical records of 82 patients of hirsutism maintained in the department were analyzed retrospectively for age, family history of hirsutism, medications and systemic diseases, menstrual and obstetric history, and associated signs of hyperandrogenism. Quantification of hirsutism was done using modified F-G score (Table 1). Investigations included were estimation of thyroid hormones, free testosterone, luteinizing hormone (LH), follicular stimulating hormone (FSH) and serum prolactin levels and ultrasonography (USG) for adrenals and ovaries. Polycystic ovarian syndrome (PCOS) was diagnosed by Rotterdam criteria: presence of two of the three elements viz. clinical or biological hyperandrogenism, polycystic ovaries and chronic anovulation.^2^ Polycystic ovaries (PCO) were diagnosed on pelvic USG by presence of ≥12 follicles measuring 2-9 mm in diameter and/or ≥10 ml ovarian volume.^3^ Clinical hyperandrogenism was defined by presence of hirsutism, acne or androgenetic alopecia.^4^ Oligomenorrhea was defined as fewer than nine menses per year.^5^

**Table 1:** Modified ferriman-gallwey semiquantitative scoring for hirsutism^1^

Sites	Grade-1	Grade-2	Grade-3	Grade-4
Upper lip	Few hairs at the outer margin	Small moustache at outer margin	Moustache extending halfway from outer margin	Moustache extending to mid-line
Chin	Few scattered hairs	Scattered hairs with small concentrations	Complete cover, light	Complete cover, heavy
Chest	Circumareolar hairs	Circumareolar hairs with mid-line hair	Fusion of circumareolar hairs with mid-line hair giving three-quarter cover	Complete cover
Upper back	Few scattered hairs	More than a few scattered hair but still scattered	Complete cover, light	Complete cover, heavy
Lower back	Sacral tuft of hair	Sacral tuft of hair with some lateral extension	Three-quarter cover	Complete cover
Upper abdomen	Few mid-line hairs	Rather more but still mid-line	Half cover	Complete cover
Lower abdomen	Few mid-line hairs	Mid-line streak of hair	Mid-line band of hair	An inverted V-shaped growth
Upper arm and thigh	Sparse hair growth affecting not more than a quarter of limb surface	More than a quarter coverage but still incomplete	Complete cover, light	Complete cover, heavy
Forearm and legs	-	-	-	Complete cover, heavy

Forearm and hand, lower leg and feet are not included in the ‘hormonal’ score and only single value is added even when hirsutism involves these extremities bilaterally. Minimum score is zero and maximum is 36. A score of 8 = no hirsutism, 8-16 = mild hirsutism, 17-25 = moderate hirsutism, 25 = severe hirsutism. Values may vary in different ethnic groups

## Results

Data of only 50 patients was complete and could be analyzed (Table 2). Age of these patients was between 13-47 (Mean 25.84 ± 8.30) years, 25 (50%) of them were in age group of 20-30 years. The maximum hirsutism F-G score was 17 with an average of 10.3 ± 2.46. Acne was found to be associated in 32 (64%) patients aged between 15-41 years. Other signs of hyperandrogenism were acanthosis nigricans in 3 (6%) and seborrhea in 2 (4%) patients respectively. Oligomenorrhea or irregular menstrual cycles was reported by 18 (36%) patients; 10 of them had PCO as well. Androgenetic alopecia was observed in 8 (16%) patients. Nine (18%) patients had history of hirsutism in first degree relatives; 2 of them showed no investigative abnormality. Eleven (22%) patients had no investigative/menstrual abnormality except for a family history of hirsutism in first degree relatives of 2 of these patients. None of the patients was obese, hypertensive, diabetic or had clinical/laboratory evidence of adrenal, thyroid or Cushing's disease. No patient had history of any drug intake.

**Table 2:** Clinico-investigative profile of 50 hirsutism patients

Case No.	Age (yrs)	F.G. score	Acne	Menstrual history	Family history of hirsutism	Free testosterone (*N* = 0.7-3.6 ng/ml)	LH: FSH *N* < 2:1	Serum prolactin *N* < 24ng/ml	Pelvic USG
1.	21	8	−	Regular	−	Normal	Normal	Normal	PCO
2.	38	8	−	Regular	−	Normal	Normal	Normal	PCO
3.	41	11	−	Oligomenorrhea	−	Normal	Normal	Normal	Normal
4.	24	14	−	Regular	−	Normal	Normal	Normal	Normal
5.	15	14	−	Regular	−	Normal	Increased	Normal	Normal
6.	19	12	+	Regular	+	Normal	Normal	Normal	Normal
7.	30	11	+	Oligomenorrhea	+	Increased	Normal	Increased	PCO
8.	23	9	−	Regular	−	Normal	Normal	Normal	Normal
9.	19	11	−	Irregular	−	Increased	Normal	Increased	PCO
10.	27	9	−	Regular	−	Normal	Increased	Normal	Normal
11.	24	11	−	Regular	−	Normal	Normal	Normal	PCO
12.	27	8	−	Regular	−	Normal	Increased	Normal	PCO
13.	25	13	+	Regular	+	Increased	Normal	Increased	Normal
14.	22	11	+	Oligomenorrhea	+	Normal	Normal	Normal	PCO
15.	31	12	−	Regular	−	Increased	Increased	Increased	PCO
16.	23	9	+	Regular	−	Normal	Normal	Normal	Normal
17.	20	8	−	Regular	−	Normal	Normal	Normal	PCO
18.	31	9	−	Oligomenorrhea	−	Normal	Normal	Normal	Normal
19.	18	9	−	Regular	−	Normal	Normal	Normal	PCO
20.	19	17	−	Regular	−	Normal	Normal	Normal	PCO
21.	18	9	+	Oligomenorrhea	+	Normal	Increased	Normal	Normal
22.	14	11	−	Regular	−	Increased	Increased	Normal	PCO
23.	38	9	−	Regular	−	Normal	Increased	Normal	Normal
24.	29	14	−	Oligomenorrhea	−	Increased	Normal	Normal	Normal
25.	21	9	−	Irregular	−	Normal	Increased	Normal	Normal
26.	47	11	−	Regular	−	Normal	Normal	Normal	Normal
27.	36	8	−	Regular	−	Increased	Normal	Normal	Normal
28.	22	15	-	Regular	−	Normal	Increased	Normal	Normal
29.	42	9	−	Regular	−	Increased	Increased	Normal	Normal
30.	40	9	−	Regular	−	Normal	Normal	Normal	Normal
31.	14	12	−	Regular	−	Normal	Increased	Increased	Normal
32.	30	12	−	Regular	−	Normal	Normal	Normal	Normal
33.	30	6	+	Regular	−	Normal	Increased	Normal	PCO
34.	27	11	+	Oligomenorrhoea	−	Normal	Normal	Normal	Normal
35.	18	16	+	Irregular	−	Normal	Normal	Normal	Normal
36.	24	8	−	Oligomenorrhoea	−	Normal	Normal	Increased	Normal
37.	17	11	−	Regular	−	Increased	Normal	Normal	Normal
38.	13	8	−	Irregular	−	Normal	Normal	Normal	Normal
39.	26	8	+	Regular	+	Increased	Normal	Normal	Normal
40.	34	12	−	Regular	−	Normal	Increased	Normal	Normal
41.	38	6	−	Irregular	−	Normal	Normal	Increased	Normal
42.	16	8	+	Irregular	−	Normal	Increased	Normal	Normal
43.	20	11	+	Oligomenorrhoea	−	Normal	Normal	Increased	PCO
44.	29	8	+	Irregular	+	Normal	Increased	Normal	Normal
45.	21	11	+	Regular	−	Normal	Increased	Normal	Normal
46.	15	13	+	Regular	+	Normal	Normal	Normal	Normal
47.	28	11	+	Regular	−	Increased	Normal	Normal	Normal
48.	36	8	+	Irregular	−	Normal	Normal	Increased	Normal
49.	29	7	+	Irregular	−	Normal	Increased	Normal	PCO
50.	23	10	−	Regular	+	Normal	Increased	Increased	Normal

F.G. Score - Ferriman-Gallwey score, + Present, − Absent, PCO - polycystic ovary, USG - ultrasonography, LH: FSH - Leutinising hormone: Follicular stimulating hormone

Serum free testosterone levels were elevated in 11 (22%) patients and 10 (20%) patients had elevated serum prolactin levels. LH/FSH ratio was increased in 17 (34%) patients, 9 of them had normal serum free testosterone levels and menstrual cycles. Fifteen (30%) patients revealed polycystic ovaries on pelvic USG. In total 17 (34%) patients fulfilled Rotterdam criteria^2^ for hyperandrogenism of polycystic ovarian syndrome origin and 9 of them also had anovulatory menstrual cycles.

## Discussion

Hirsutism affects 5-10% of women of reproductive age.^1^ It can be classified as androgenic and non-androgenic hirsutism. Hirsutism can be caused by abnormally high androgen levels or by hair follicles which are more sensitive than usual to normal androgen levels. Biologically active free testosterone is responsible for hair growth and is regulated by sex hormone-binding globulin. The causes of androgenic hirsutism can be exogenous due to drugs (testosterone, dehydroepiandrosterone sulfate, danazol, corticotropin, high-dose corticosteroids, metyrapone, phenothiazine derivatives, anabolic steroids, androgenic progestin, and acetazolamide) or excess endogenous androgen of adrenal or ovarian origin. Various causes of ovarian hyperandrogenism are PCOS and virilizing ovarian neoplasia (Luteoma of pregnancy, arrhenoblastomas, leydig cell tumors, hilar cell tumors, thecal cell tumors, etc.). However, PCOS alone accounts for 75-80% cases of hyperandrogenism.^4^,^6^ Clinically the most common sign of hyperandrogenism in PCOS is hirsutism. The prevalence of hirsutism in PCOS varies between 17% and 83%.^7^ Polycystic ovaries were detected in our 30% patients. However by using Rotterdam criteria for diagnosing PCOS,^2^ 17 (64%) of our patients had clinical hyperandrogenism. Acne appears another consistent feature and was found in 64% patients across all age groups followed by oligomenorrhea in 18% and androgenetic alopecia in 16% patients respectively. Acanthosis nigricans (6%) and seborrhea (4%) appear other but less common signs of hyperandrogenism in our patients. Since gonadotrophins are released in a pulsatile manner their concentration varies over the menstrual cycle and a single measurement of LH and/or FSH may not be a sensitive method for diagnosis.^5^ This is also evident in our 17 patients having abnormal LH to FSH ratio but elevated serum free testosterone levels and abnormal menstrual cycles, features of hyperandrogenism, were observed in 11 and 9 patients only.

Adrenal hyperandrogenism is uncommon and seen in congenital adrenal hyperplasia, late-onset adrenal hyperplasia, Cushing's syndrome, pituitary adenomas that produce excess corticotropin or prolactin and acromegaly. None of our patients had adrenal abnormality. Hirsutism in 8 of 10 patients having raised serum prolactin levels appears to be more of PCOS associated hyperandrogenism in view of additional features such as elevated free serum testosterone, LH-FSH ratio, oligomenorrhea and/or polycystic ovaries.

The non-androgenic causes of hirsutism may be familial, idiopathic or due to drugs like cyclosporine, phenytoin, diazoxide, triamterene-hydrochlorothiazide, minoxidil, hexachlorobenzene, penicillamine and psoralens. Other less common causes include anorexia nervosa, hypothyroidism and porphyria. Idiopathic hirsutism, also called simple or peripheral hirsutism, is diagnosable in women who have normal ovulatory function and normal androgen profile. Only 5-15% of hirsute women qualify for this diagnosis by these criteria.^4^,^7^,^8^ Except for our two patients who had hirsutism in first degree relatives other 4 patients with normal investigative profile can be considered to be of idiopathic origin.

Despite limitations of small number of patients and lack of long-term follow-up in most patients, the study indicates that hirsutism in Indian women is not uncommon. Although its clinical presentation does not differ from its description in the literature, hirsutism of adrenal or thyroid origin does not appear to be common in our patients. Interestingly, while patients in the adolescent group showed more concern for their facial hair than acne, the patients in the older age group had worries more often for late onset acne signifying a varied perception of the same problem. However, all patients were more concerned for facial hair than those on other body areas. We feel that facial hair be given higher than current value in F-G scoring system in view of the psychological/cosmetic embarrassment it causes.
